# Counseling for personal care options at neonatal end of life: a quantitative and qualitative parent survey

**DOI:** 10.1186/s12904-015-0063-6

**Published:** 2015-12-02

**Authors:** Emily Shelkowitz, Sharon L. Vessella, Patricia O’Reilly, Richard Tucker, Beatrice E. Lechner

**Affiliations:** Department of Social Work, Women and Infants Hospital, Providence, RI USA; Warren Alpert Medical School of Brown University, Providence, RI USA; Department of Pediatrics, Women and Infants Hospital of Rhode Island, 101 Dudley St, Providence, RI 02905 USA

**Keywords:** Neonate, Death, Memory making

## Abstract

**Background:**

The death of a newborn is a traumatic life changing event in the lives of parents. We hypothesized that bereaved parents of newborn infants want to have choices in the personal care of their infant at the end of life.

**Methods:**

Parents who had suffered a perinatal or neonatal loss between 1 and 6 years before the survey in a regional level IV neonatal intensive care unit (NICU) and associated labor and delivery room were invited to participate. Parents chose between an online survey, paper survey or telephone interview. The survey included multiple choice and open ended questions.

**Results:**

Parents prefer multiple options for the personal care of their infant at the end of life. Emergent themes were need for guidance by the medical team, memory making, feeling cared for and respected by staff, and regrets related to missed opportunities.

**Conclusion:**

While parents differ in their preferences in utilizing specific personal care options for their infant’s end of life, they share a common preference for being presented with multiple options to choose from and in being guided and supported by healthcare providers, while being afforded the opportunity to make memories with their infant by bonding with and parenting them.

**Electronic supplementary material:**

The online version of this article (doi:10.1186/s12904-015-0063-6) contains supplementary material, which is available to authorized users.

## Background

Four out of 1000 live births in the United States end in neonatal death before 28 days, while three out of 1000 pregnancies end in stillbirth [1]. The experience of stillbirth or neonatal death may lead to psychological sequelae in parents, including anxiety, post-traumatic stress disorder and depression [2–5]. Medical providers at the time of stillbirth or death are in a unique position to offer support and guidance in the immediate grieving process, as parents’ ability to grieve the loss of their child depends not only on pre-existing factors such as marital quality, mental health and social support before the loss [6], but also on the approach of their medical providers. How caregivers interact with parents greatly impacts their experience of stillbirth or their infants’ end of life.

Provider-parent communication and decision-making play a key role in determining the degree to which parents are satisfied with the care that they and their baby received, as well as in parents’ long-term grief response [7, 8]. A study of neonatal units in England found that professionalism, which included information being provided and explained to the parents in a calm, confident and controlled manner, and empathy, which included being caring and providing emotional support, encouragement and reassurance, were crucial determinants in the level of parental satisfaction with neonatal intensive care unit (NICU) staff [9]. Likewise, a systematic review of articles addressing fetal death and early infant loss from 1966 to 2006 found that avoidance, insensitivity and poor staff communication were most distressing to parents [10]. Finally, a study of end-of-life decision making in the NICU has shown that parents experience less grief when they share the responsibility of decision making with the physician rather than when decisions are made without involvement of physicians beyond the transmission of clinical information or when decisions are made by the physician without taking into account parental opinions/concerns [8].

In addition, parents’ ability to bond with their baby and parent them in the NICU is strongly dependent on staff interactions with the parents and their support in facilitating the bonding [11]. Furthermore, a prominent theme for parents after stillbirth is “making irretrievable moments precious” [12]. At their infants’ end of life, families consider memory making to be of central importance [13]. Memory making is extremely important to families who suffer a stillbirth and it is important to them to have staff encourage them and offer choices [14]. In fact, how providers manage the postpartum period and whether memory making occurs influences maternal long-term psychological outcomes [2].

At the same time, little is known about what parents are routinely offered for memory making options and how they feel about the choices they are given. Thus, the objective of this study was to explore the personal care options offered to parents as well as parental perceptions of the counseling they received regarding these options at their infants’ end of life in order to identify approaches to improving the parental experience of a stillbirth or neonatal death.

## Methods

### Study design

We performed a cross-sectional survey study of mothers who had suffered a stillbirth or neonatal loss (defined as live birth of any gestational age or stillbirth over 20 weeks gestation) in the labor and delivery unit or the 80-bed regional level IV neonatal intensive care unit of a regional medical center between one year and five years prior to the study (January 1, 2009 to December 31, 2012). This time range was chosen to allow parents at least one year to grieve their loss, but not long enough for the accuracy of recall to suffer. These parents were identified through review of medical records and contacted for recruitment via letter. The number of parents identified as having suffered a stillbirth or neonatal loss during the study time period was 413. Eight parents were excluded because they had previously participated in similar studies, as per Institutional Review Board requirement. There was no known address or the request to contact letter was returned as unknown for 87 parents. A response to the request to contact letter, which included a stamped return opt-in/out card, was not received from 275 parents. Three parents declined request to contact via opt-in/out card. Twelve parents who opted in and received the survey did not complete the survey. The number of parents who responded to the survey was 28 (Table 1). Consent was obtained from these participants. Institutional Review Board approval was obtained for the study. As per Institutional Review Board requirement, parents who did not return the opt-in/out card were not sent reminders.

**Table 1:** Neonate characteristics

Characteristic	Number of infants (*n* = 28)
Gestational Age, wks (SD)	28.6 (6.8)
Male, %	62.5
Time elapsed since death, yrs (SD)	2.8 (1.2)
Parity	
Singleton	24 (86 %)
Twins	3 (11 %)
Triplets	1 (3 %)
Diagnosis	
Congenital^1^	3 (11 %)
Genetic^2^	2 (7 %)
Prematurity	12 (43 %)
In utero fetal demise	8 (28 %)
Other	3 (11 %)

^1^Congenital malformations: malformations not associated with documented chromosomal abnormalities

^2^Genetic syndromes: documented chromosomal abnormalities

The following demographic and clinical data were obtained on respondents via retrospective chart review: maternal age, race, ethnicity, marital status, insurance status, gestational age, gender, diagnosis and time elapsed since the death. Additionally, the following demographic data were obtained from non-respondents: maternal age, race, ethnicity, marital status and insurance status. Non-respondents were all parents identified who met clinical criteria within the study timeframe for whom contact information was available plus parents with no known address or with returned letters.

### Survey design

The survey on the circumstances and contents of personal care options counseling of parents whose infant died in the labor and delivery room or the NICU was developed by the study team based on clinician expertise. The survey consisted of 24 multiple choice questions as well as the option to describe their experience with the death of their infant in their own words. The survey included the following domains: place, timing and person of counseling, specific topics of counseling and the respondents’ utilization of the options of counseling, as well as the respondents’ understanding of their infants’ condition. A multidisciplinary group of neonatal healthcare providers (the NICU palliative care taskforce, including physicians, nurses, social workers and chaplains) reviewed the survey for question content and goals. The survey was sent to two volunteer parents of children who had died for pilot testing and feedback. The survey was modified to include parent and taskforce feedback. The survey was not validated. The survey questions are listed in Additional file 1.

### Survey procedure

Parents chose whether to complete the survey electronically (via SurveyMonkey, Palo Alto, CA), on paper, or over the telephone. One of the telephone respondents was a Spanish speaker and was thus surveyed over the phone by a native Spanish speaking neonatologist. All other respondents completed the survey in English. The survey questions related to the personal care options offered to parents at the end of their infant’s life. Among the specific personal care options surveyed, keepsakes were defined as items such as memory boxes put together by staff, hand- and footprints, locks of hair, the infant’s hospital hat, blanket and identification bracelet, and similar items. Support person refers to sources of support who are present for the patient/mother, included husband, family or friends. “Now I Lay Me Down to Sleep” refers to a volunteer service consisting of professional photographers who take pictures of dying or deceased babies/children and their families in the hospital. Special clothing/blanket refers to items that the family could choose to bring in to dress the baby. Private birthing/c-section classes are prenatal educational sessions provided to families who have received a lethal prenatal diagnosis, so that they can attend a prenatal class tailored to their unique needs and without the need to be surrounded by couples with healthy pregnancies. Skin-to-skin refers to skin-to-skin care, in which parents hold their baby on their bare chest.

### Data analysis

Electronic, paper and telephone interview results were captured and stored in a de-identified password protected database. Quantitative data analysis was performed using SAS 9.1 (Cary, NC) statistical software. Continuous variables were analyzed by t-test, or Wilcoxon test in the case of a non-normal distribution. Categorical data was analyzed by the chi-square test. Qualitative data were collected and analyzed for prevalent themes/patterns by the study team. Quotes are verbatim except where identifying information was deleted or changed to protect confidentiality without changing the meaning of the statement. The authors performed content analysis. The main themes that emerged from this initial analysis were then used to code each statement by parents into the theme categories. Additional independent content experts from the NICU palliative care group ratified the coding.

## Results

**Fig. 1:**
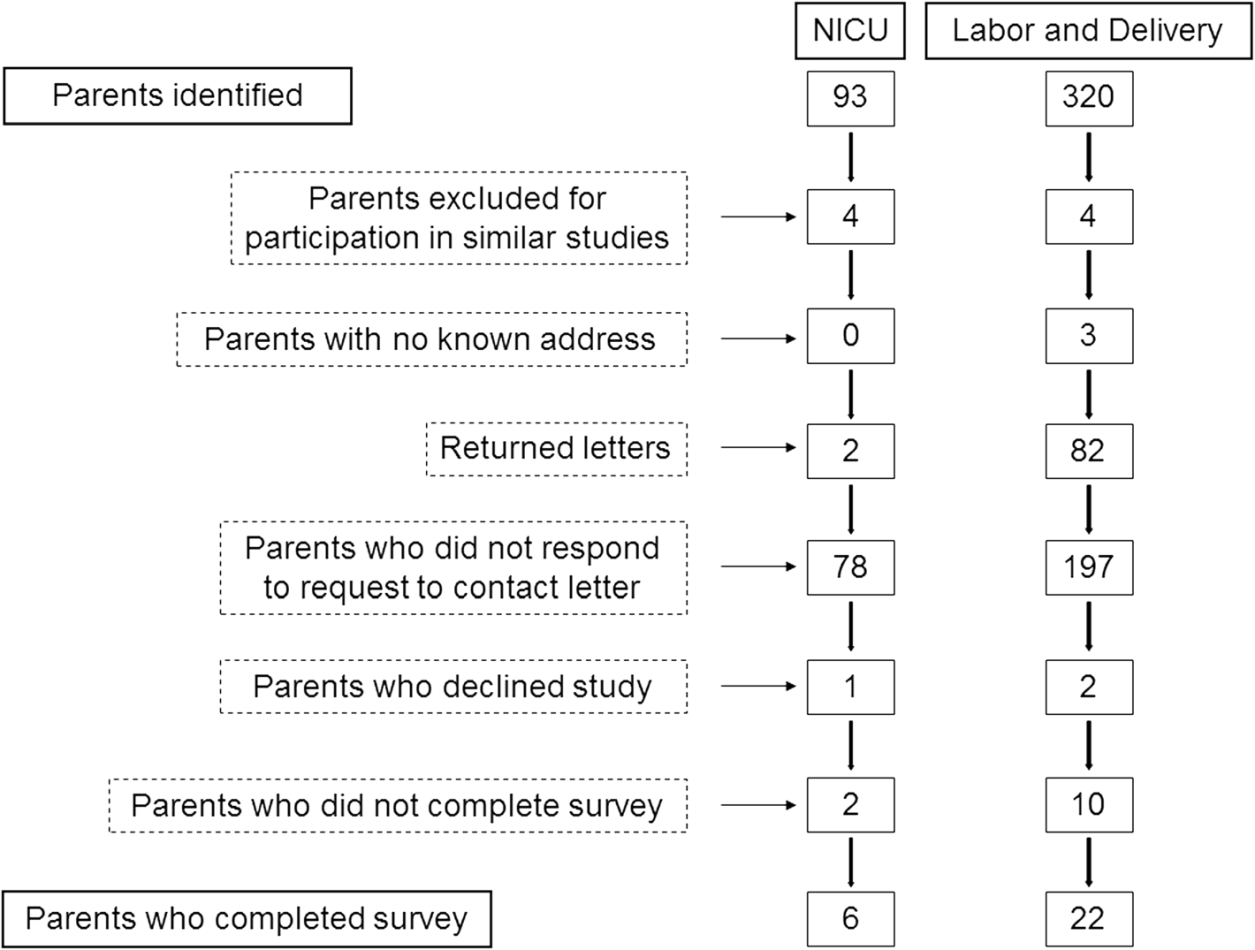
Survey respondent breakdown. Response rate: 10 % of all parents identified for whom contact information was available; 70 % of parents who agreed to be contacted and who subsequently received the survey. NICU = neonatal intensive care unit

Six parents of infants who died in the NICU and 22 parents of infants who died in utero or in the labor and delivery room without admission to the NICU responded to the survey. The response rate was 10 % of all parents identified for whom initial contact information was available. Of parents who agreed to be contacted (those who opted in) and who subsequently received the survey, the response rate was 70 % (Fig. 1).

Neonatal characteristics of respondents are listed in Table 1. Most surveys were completed in the electronic format (*n* = 16), while 8 were completed in the paper format and 4 per telephone interview. In most cases (*n* = 27), the mother completed the survey. No fathers completed the survey. In one case, the mother and father completed the survey together.

**Fig. 2:**
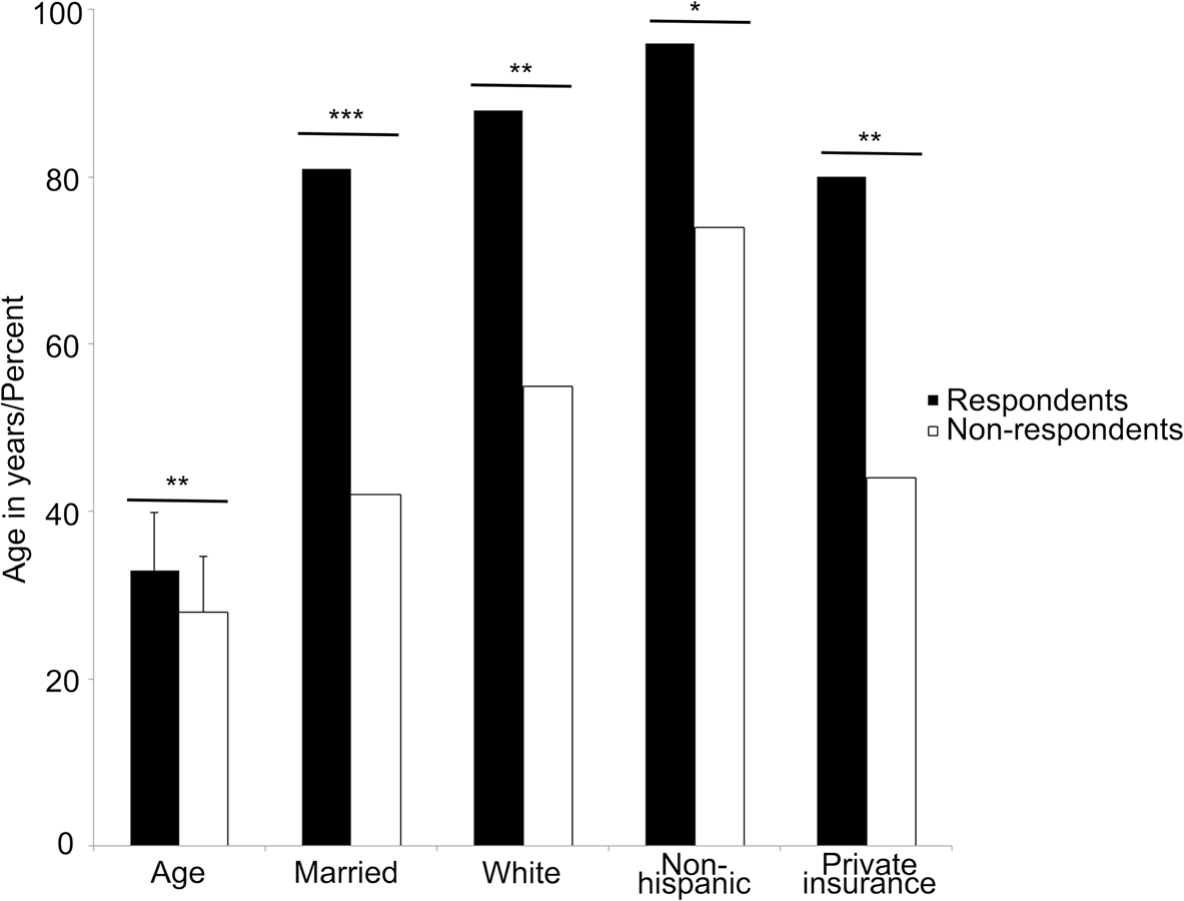
Maternal demographics. Mean maternal age and percentage of mothers who are married, white, non-Hispanic, and have private insurance in the respondent group compared to the non-respondent group. Each demographic variable is significantly increased in the respondent group. Maternal age, p = 0.002; Married, p < 0.001; Race, *p* = 0.002; Ethnicity, *p* = 0.02; Private insurance, *p* = 0.004. Student’s T-test and χ^2^ test

Study respondent demographics were different than non-respondents. Respondents were older (*p* = 0.002, mean 33 years, range 17–45 years, compared to non-respondent mean of 28 years) and more likely to be white (*p* = 0.002), non-Hispanic (*p* = 0.02), married (*p* < 0.001) and to have private insurance (*p* = 0.004) compared to non-respondents (Fig. 2).

In the group whose infants died in the labor and delivery suite, before admission to the NICU, parents who received counseling about the personal care options available to their infant usually received this counseling during the visit or hospitalization when the diagnosis was made, with most counseling occurring after the infant was born.

**Fig. 3:**
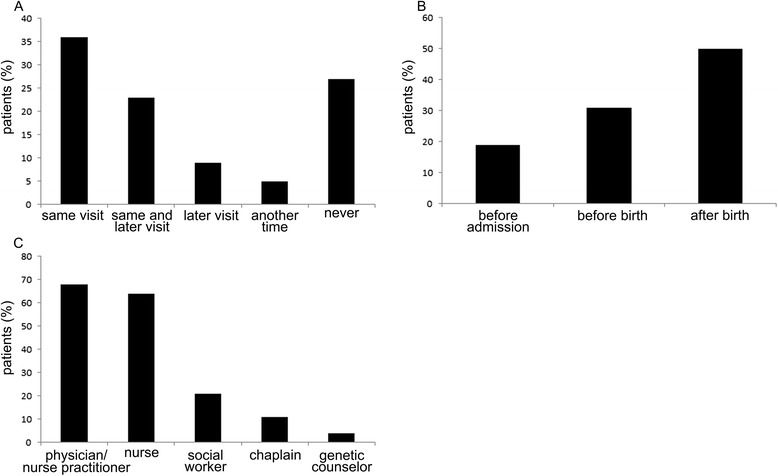
Counseling on available personal care options offered to parents. **a** Timing of counseling in relation to timing of diagnosis. In most cases, counseling started at the same visit, but a significant number of parents did not receive personal care option counseling at all. **b** Timing of counseling in relation to timing of birth. Most counseling occurred after birth. **c** Person who provided counseling. Most counseling was provided by physicians and bedside nurses

Parents were asked about the timing of the counseling they had received on the personal care options available to them at the end of their infant’s life. 36 % of parents responded that they had received this counseling during the same visit or hospitalization in which they learned their infant’s diagnosis or prognosis. 23 % of parents received this counseling during both the initial visit and at later visits. 9 % of parents received this counseling during a later visit/hospitalization and 5 % of parents received personal care option counseling at another time. 27 % of parents stated that they had received no counseling on personal care options (Fig. 3a). Of those that reported having received counseling, 19 % received this counseling before admission to the hospital, 31 % received it in the hospital before birth and 50 % was counseled in the hospital after the birth of their infant (Fig. 3b).

Of those that received counseling, 77 % thought this counseling was provided at the appropriate time. Of those that did not receive counseling on the personal care options available to them, 60 % would have preferred to have received counseling.

Most of the counseling was performed by physicians or nurse practitioners, followed closely by nurses, with a smaller component of counseling performed by social workers, chaplains and genetic counselors (Fig. 3c).

**Fig. 4:**
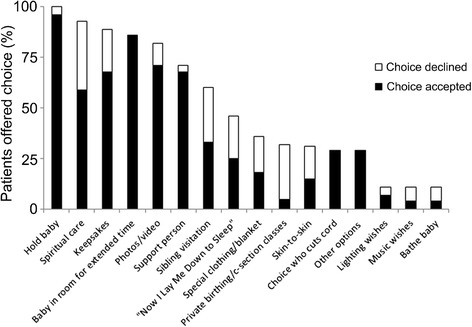
Personal care options offered and used. The incidence of personal care options offered to parents at the end of the infant’s life varied, as well as the incidence of parents taking advantage of each option offered. Keepsakes are memory boxes, hand- and footprints, locks of hair, etc. Support person is husband, family or friends who are present. “Now I Lay Me Down to Sleep” is a volunteer service of professional photographers. Special clothing/blanket are items that the family chooses to bring in to the hospital. Private birthing/c-section classes are prenatal educational sessions offered in the setting of a lethal prenatal diagnosis. Skin-to-skin is parents holding their baby on their bare chest

The incidence of specific personal care options being offered to parents displayed a wide range. At one end of the spectrum, caregivers offered the mother the option to hold her baby 100 % of the time, while at the other end of the spectrum, options for music or the opportunity to bathe the baby were rarely offered (11 %). Also, there was variability in how often individual options were utilized by parents, ranging from 100 % percent utilization of the option to choose who cuts the umbilical cord and to keep the baby in the room for an extended period of time after death, to 17 % utilization of private prenatal birthing/cesarean section classes (Fig. 4).

**Fig. 5:**
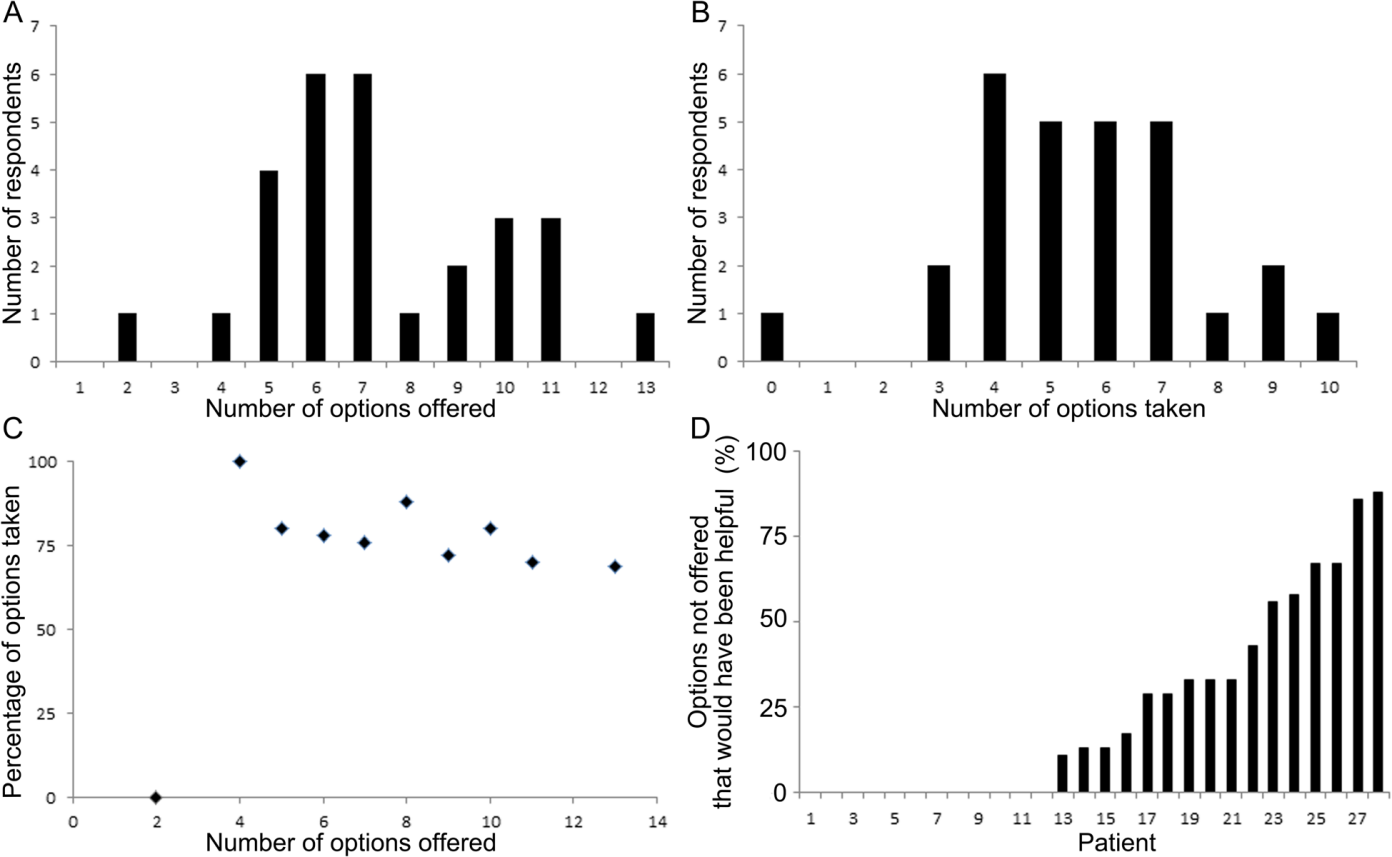
Number of personal care options offered and taken. **a** Number of options offered. 7.4 options were offered to each family on average. **b** Number of options taken. 5.6 options were utilized by each respondent on average. **c** Most offered options were utilized. An average of 75 % of options offered to a family were utilized, with 96 % of respondents utilizing at least 69 % of options offered. **d** Percent of options not offered that would have been helpful. 57 % of respondents felt that at least some things were missing that could have been offered. On average, 24 % of options not offered would have been helpful had they been offered

Parents preferred having options to choose from and took advantage of multiple options for the personal care of their infant. On average, 7.4 options were offered to each parent (Fig. 5a) and 5.6 of the options offered were then utilized by the parents (Fig. 5b). 96 % of parents utilized at least 4 infant personal care options, or at least 69 % of the options offered to them. On average, 75 % of personal care options that were offered were utilized, with 96 % of respondents utilizing at least 69 % or at least 4 options offered, irrespective of whether fewer or more options were offered to a parent (Fig. 5c). 57 % of respondents felt that at least some things were missing that could have been offered. On average, respondents felt that 24 % of options not offered would have been helpful had they been offered (Fig. 5d). Conversely, only one respondent indicated that they would have preferred not to have been offered one option that was offered (data not shown).

29 % of respondents received additional options not listed. Of these patients, 100 % felt that receiving these additional options was helpful.

79 % of respondents felt that the number of options they received was just right. None felt they received too many, and 21 % felt they received too few.

Parents experienced specific personal care options offered to them at the end of their infant’s life in varied and individual ways.

### The presence of a support person

While some mothers would have liked an extended support network in the room, others chose not to utilize this option. Comments ranged from *“We had chosen to have only my husband and myself in the labor room for the birth… We opted not to have family with us”* to *“It would have been nice to know that the grandparents could have been there.”*

### Sibling visitation

Sibling visitation was offered as an option in 60 % of cases in which there was a sibling. Of these, 56 % visited. Some mothers stated that they wanted their older children to meet their sibling, while others specifically chose not to do that. *“I wanted my husband and I to grieve alone personally and then as a united front explain to the kids”. “It was important for us to grieve as a family and allow our children to see their sibling.” “My older daughter did come and visit with her sister. To this day I am not sure it was the right decision.”*

### Spiritual care

While some families found spiritual support very important for their grieving process, others experienced it as a burden. *“It was good to have pastors pray and encourage us.” “Family brought a spiritual faith person into the room, we did not appreciate hearing about God’s plan after watching our otherwise healthy twins pass away from early labor.”*

### Special music/lighting

Parents did not have preferences for specific lighting or music. *“…lighting…would have been irrelevant to us at the time given the circumstances.” “I was focused on the baby and my husband. I didn’t care about lighting, bedding or anything else.” “… I am glad there is no song that I will hear on the radio that I would associate with my son’s death.”*

### Photographs

Most parents cherish having photographs, but the sentiment was not universal. *“Photos of a dead baby would take away from the spiritual image that was more important to me.” “We are so grateful to “Now I lay me down to sleep” photographers. We went so unprepared but now we have pictures to cherish.”*

Respondent preferences did not differ depending on place of death, the timing of counseling, whether parents knew that their infant may not survive, or religious or spiritual beliefs. 68 % of parents knew there was a chance that their baby would not survive, while 32 % were not aware of this. Parents who knew and those who did not know did not differ in their preference for options or their utilization rate of options (data not shown). Preference for options or the utilization rate for options also did not depend on when counseling occurred or whether the infant died in the labor and delivery room or the NICU (data not shown).

94 % of respondents self-identified as religious, 75 % as spiritual, and 87 % as either religious or spiritual. There were no differences in these groups in terms of preference for options or their utilization rate of options (data not shown).

Qualitative data was grouped according to recurrent themes. Four dominant themes emerged from the respondents’ descriptions of important aspects of the parental experience of losing their infant.

1. Guidance by healthcare providers
  Parents addressed the necessity of their healthcare providers providing guidance. They described the importance of receiving information to assist with decision making by being presented choices and options, given that this was a very difficult time and they were not prepared with the knowledge of what reasonable options are and what would be helpful to them in their long term grieving process.
  *“I was encouraged by the nurses to change her diapers and take her temperature, which was difficult but looking back I am grateful for that”. “We were glad it was offered, it just wasn’t for us.” “It is a very confusing and heartbreaking time and it would be nice to see these options offered.”*

2. Making memories
  The importance of making memories was described by many mothers, since memories are all that parents can take away with them to remind them of the short amount of time they shared with their infant and had the opportunity to parent their infant. Parents noted that they appreciated having a memory box, as well as mementos such as handprints, footprints, locks of hair, photos or having held their infant.
  *“We held him for a long, long time. The nurses were so understanding and did not rush us.” “I was so overwhelmed with the situation that I barely remember the day, but having his pictures and knowing that my husband and I both held him (even though we were nervous to at first) means the world.” “The memory box allows me to have a piece of my baby for the future. It reminds me that he was real and what my husband and I went through was real. It helps.”*

3. Feeling cared for and respected
  Parents described the importance of feeling cared for and respected by the medical team and nursing staff during this difficult time in their life.
  *“Everyone was so caring respectful and felt our sadness with us.” “I will be forever grateful to the hospital for their many kindnesses to my family during such a difficult time. So many were so caring and kind.” “When we were ready to leave the room, someone came in the room for the baby’s body and started to put him in a bag in front of us. We felt it was disrespectful and the body should have been carried out of the room or it should have waited until after we left. We did not want to see that. All other aspects of our hospital stay were handled appropriately and respectfully.”*

4. Regrets/wishes
  Some parents described feelings of regret for what they wished had happened differently or that they could have done differently. Among those, the longing to have spent more time with the baby and to have made more memories with their infant was a significant recurrent theme that was the most prevalent theme of the qualitative questions.
  *“I did get his blanket and hat but wished I had footprints and handprints.” “I would have liked to do this* [bathe the baby]*, it did not occur to me to look at his whole body, he was swaddled and later I had wished I had looked at every inch of him.” “I really wish I had spent time with the twins while they were still alive and breathing. I wish I had been told that they were breathing.”*

## Discussion

In this study we show that counseling on the personal care options available to families for their dying or stillborn infant is performed early in relation to the lethal diagnosis being made, but usually after birth. Providing this information to families in a timely manner is important on multiple levels. First, good communication with their child’s healthcare providers is a very important aspect for parents when their children are dying [15, 16] and parental perceptions of healthcare providers’ communication influence long-term parental grief levels after the death of their child [17]. Second, when parents are confronted with an unfamiliar and stressful situation, they rely heavily on guidance by the medical team. The importance of memory making with their infant is not only one of the four major themes that emerged in our family interviews, but missed opportunities to make additional memories was also the predominant component of parental wishes and regrets. Others have also shown memory making to be of utmost importance to families at the end of their infant’s life [13, 18]. Nonetheless, over a fourth of patients did not receive counseling on the personal care options available to their infant, and almost two thirds of those who didn’t would have appreciated receiving counseling. While these numbers may be altered secondary to recall bias, and in some cases the specifics of the clinical scenario may have precluded counseling, the numbers nonetheless suggest an opportunity for the implementation of additional safeguards to ensure counseling.

Our results suggest that rather than being overwhelmed by options for the personal care of their infant at the end of life, parents appreciate being offered options even if they ultimately choose not to utilize every one offered to them. Given that infant death is no longer the societal norm it once was [19], and that parents are in a state of crisis, most parents are not aware of the importance of bonding and memory making for their long term grief/bereavement process, nor are they usually aware of the available options to parent their infant and make memories. Thus, it is important that healthcare providers offer sufficient options. Our study demonstrated a wide range in the number of options offered to each parent, as well as a wide range of options utilized by parents. It is interesting to note that some options, such as holding the baby, were offered to all parents, possibly because of the strong evidence of the utility of this practice. For example, being afforded the opportunity to bond with a dying baby is imperative for grieving and the lack of opportunity to do so leads to complicated grief and mental health issues [20, 21]. In fact, parents who were handed their infant to hold without being asked if they wanted to hold the baby reported feeling less frightened and more comfortable than parents who were asked [22]. On the other hand, certain options were rarely offered, such as bathing the baby, possibly because knowledge of this option requires advanced skills in infant end of life care. Thus, more education in infant end of life care and memory making may be warranted.

Similarly, some options were not as commonly utilized by families as other options. The option to hold the baby was utilized by most parents and the option to keep the baby in the room with the mother for an extended period of time was utilized by all parents. In fact, studies have demonstrated for decades that grieving mothers want to be able to see their infant for an extended period, sometimes even several days after the death [23]. At the same time, some options were less commonly utilized. Among these were the options surrounding having a support person in the room and who that support person should be, the option of sibling visitation, as well as the involvement of spiritual care. The utility of each of these options is heavily dependent on interpersonal relationships as well as individual beliefs; hence the variability in utilization is expected. The options of lighting and music wishes were also not often utilized. While healthcare providers and families agree that the physical environment plays a role in the provision of appropriate end of life care [24, 25], and many parents likely appreciate an environment of soft, soothing lighting as opposed to harsh clinical lights, the authors surmise that healthcare providers often routinely make the adjustment without consulting the family, leading to parents not being consciously aware of lighting adjustments as an “option” which they can (or want to) think about. While music therapy is an important component of pediatric palliative care [26], which has been shown to reduce stress in NICU patients [27], it may not be a concept many parents are aware of as a medical intervention and thus they may question the utility or the appropriateness of it in the setting of end of life care and grieving.

Our data suggest a difference in utilization rates of options such as holding the baby and spending additional time with the baby, which may be regarded as necessary options that enable bonding, parenting and the creation of memories, compared to the other aforementioned options that constitute stylistic approaches to bonding, parenting and the creation of memories that greatly depend on interpersonal relationships, cultural, religious and other beliefs that vary among individual parents.

Additionally, the one third of mothers who were offered additional options all chose to utilize those options. A likely interpretation of this finding is that the offering of additional options reflects the healthcare provider tailoring additional options to the patient and their specific needs beyond the most common options offered.

The fact that 79 % of parents felt that the number of options they received were just right may be a reflection on the healthcare providers providing appropriate care. Alternately, it may mean that parents often experience their own situation as normative without the means to compare, so whatever they were offered seemed appropriate to them. The fact that none of the parents felt that they were given too many options supports the conclusion that parents prefer to have options, while the 21 % who received counseling on too few options indicate that additional counseling in these difficult situations may be helpful for many patients. The theme of regrets/wishes that emerged from the survey further underlines this conclusion.

Despite variability in the utilization of individual personal care options, there is consistency among parents on the overarching themes, including how they felt about the number of options offered and how many options they utilized irrespective of additional factors such as whether they knew their infant may not survive, the timing of counseling, the location of death or the spiritual or religious self-identification of the parent.

The major themes that emerged from the survey reveal that bonding with and parenting their baby, as well as making memories, is a central component of the experience for parents. Furthermore, they look to their healthcare providers for guidance on how to accomplish this, and expressed wishes and regrets related to what did not occur in terms of memories and bonding. These findings are consistent with research suggesting that parents want options for memory making and encouragement from healthcare providers if they are initially hesitant, and regret what they did not do, for example in the realm of bereavement photography [28]. Additionally, they expressed the need to feel supported, respected and nurtured in the process. Thus, our results suggest that the guidance parents receive from the healthcare team on personal care options available to them has a great impact on the parental experience.

Importantly, the demographics of parents who responded to the invitation to participate in the study were significantly different than non-respondents. The respondents were older and more likely to be white, non-Hispanic, married and to have private insurance, as well as being mothers, not fathers. This is an important caveat to our findings, which mirrors respondent profiles of similar studies [29, 30], and begs the question of how to capture responses from the more diverse population. Demographic and patient-specific factors need to be considered in addressing the question of how the clinical approach can be modified to improve the parental experience by respecting the needs of all demographic groups. For example, a pilot study suggests that African- American parents are more likely to desire the continuation of life-sustaining-measures at the end of life than white parents [31] and more Hispanic and black mothers than white mothers had moderate or severe depression and post-traumatic stress disorder during the first year after their infant or child’s death in the NICU or PICU [3]. Furthermore, marital quality, mental health and social support before the loss are all correlated with parental grief [6], suggesting that the non-respondent group, based on their demographics, might be at increased risk. Thus, a future direction of research should be aimed at addressing the experiences of the diverse non-respondent population.

The response rate of 10 % of all eligible parents is low. However, such response rates are expected in pediatric end of life studies utilizing the opt-in method of recruitment as opposed to the opt-out method [32]. Some Institutional Review Boards, including ours, require multiple steps for bereaved parents to opt-in to research studies. This practice of opt-in vs opt-out recruitment results in lower response rates, for example 12 % vs 30 % in a study of bereaved parents [33] or 16 % vs 54 % in a public health study [34]. However, of parents who opted in and thus received the survey, the response rate was 70 %. In terms of actual numbers of patients recruited (n = 28), this study is within the norm compared to recent studies with bereaved parents in which 11 parents were interviewed in one study [35], the parents of 5 children who died were interviewed in another [36], and the parents of 17 children who died were interviewed in a further study [37]. Additionally, there was a wide range in gestational age, parity and neonatal diagnosis, as well as in parental responses, suggesting sufficient variation in parental experience.

While this study addressed the content of the counseling on personal care options, an important aspect of such counseling that was not addressed in detail in this study is the quality of the actual communication during the counseling session [38, 39]. Whether evidence based communication skills for difficult conversations were used and whether their uniform use may influence the results of such a study was not addressed in this study but is a very important aspect for future study.

Another aspect to consider when interpreting this data is that the two locations in which the surveyed parents experienced their loss were quite distinct. The typical diagnosis is different in deaths occurring in the NICU and the labor and delivery room, thus the clinical scenario and the timeline are often different. A situation in which parents learn of a lethal diagnosis and receive counseling in the outpatient setting is very different from preterm premature rupture of fetal membranes at a previable gestational age with an extended period of induction of labor in the labor and delivery room, compared to a premature feeding and growing infant in the NICU who develops fulminant necrotizing enterocolitis. In addition to the range in clinical scenarios, each family is unique in terms of their ethnic, cultural and religious background, their social environment, mental health and support network. Thus, a discrete yet broad-based approach to the personal care options counseling of each individual family is necessary. As one respondent astutely noted, *“Every situation is unique. What services are offered to some may not work for others.”*

## Conclusions

In summary, while parents who responded to this survey differ in their preferences in utilizing specific personal care options for their infant’s end of life, this study is novel in that it demonstrates that these bereaved parents share a common preference for being presented with multiple options to choose from and in being guided and supported by healthcare providers, while being afforded the opportunity to make memories with their infant by bonding with and parenting them. These observations are important in designing interventions to suit the unique needs of the bereaved families of infants.

## Additional file

Additional file 1:
**LDR survey and NICU survey.** Survey questions and response options. (PDF 326 kb)

## Abbreviations

NICU: Neonatal intensive care unit
