# Supplementary material for: Counseling for personal care options at neonatal end of life: a quantitative and qualitative parent survey
Source: BMC Palliat Care. 2015 Dec 2;14:70. doi: 10.1186/s12904-015-0063-6 (PMC4667527; doi:10.1186/s12904-015-0063-6)
Supplement: Additional file 1: — LDR survey and NICU survey. Survey questions and response options. (PDF 326 kb) [file 12904_2015_63_MOESM1_ESM.pdf]

LDR survey

After receiving news about your baby's diagnosis or test results, when did you receive counseling regarding options for how you would like to spend the time you have with your baby?

☐ During the same visit/conversation → Was this a good time to talk about this? ☐ Yes ☐ No
☐ At a later visit/conversation → Was this a good time to talk about this? ☐ Yes ☐ No
☐ Both
☐ Not at all → Would you have liked to be counseled? ☐ Yes ☐ No
Additional comments:____

When did you receive this counseling?

☐ Before admission to the hospital for delivery
☐ In the hospital before delivery
☐ In the hospital after delivery
☐ I did not receive counseling regarding options for how I would like to spend the time I have with my baby

From whom did you receive counseling regarding options for how you would like to spend the time you have with your baby? Please choose all that apply:

☐ Obstetrician or maternal/fetal medicine (perinatology) doctor at Women & Infants
☐ Neonatologists at Women & Infants
☐ Your own obstetrician
☐ Your own pediatrician
☐ Genetic counselor
☐ Nurse in the labor and delivery room
☐ Social worker
☐ Hospital chaplain
☐ Other ______________________________________
☐ Did not receive counseling regarding options for how I would like to spend the time I have with my baby

Was it discussed with you that you could choose to have family members or other sources of support in the delivery room?

☐ Yes → Did you have a support person? ☐ Yes ☐ No
Would you have preferred not to be offered this choice? ☐ Yes ☐ No
☐ No → Would this information have been helpful? ☐ Yes ☐ No
Additional comments:____

Did your baby have older siblings?

☐ Yes → Was sibling visitation discussed with you? ☐ Yes → Did the sibling(s) visit? ☐ Yes ☐ No
Would you have preferred not to be offered this choice?
☐ Yes ☐ No
☐ No → Would this information have been helpful to you?
☐ Yes ☐ No
☐ No
Additional comments:____

Were you offered spiritual care? (Either someone from your own faith or a hospital-based chaplain?)

☐ Yes → Was a spiritual care giver present at any point during your baby's life? ☐ Yes ☐ No
Would you have preferred not to be offered this choice? ☐ Yes ☐ No
☐ No → Would this information have been helpful to you? ☐ Yes ☐ No
Additional comments:____

Do you identify yourself as religious?
☐ Yes
☐ No

Do you identify yourself as spiritual?
☐ **Yes**
☐ No

Were you told you could hold your baby?
☐ Yes → Did you hold your baby? ☐ Yes ☐ No
Would you have preferred not to be offered this choice? ☐ Yes ☐ No
☐ No → Would this information have been helpful to you? ☐ Yes ☐ No
Additional comments: ____

Were you told you could hold your undressed baby on your chest (skin-to-skin care) as a treatment option?
☐ Yes → Did you do skin-to-skin care with your baby? ☐ Yes ☐ No
Would you have preferred not to be offered this choice? ☐ Yes ☐ No
☐ No → Would this information have been helpful to you? ☐ Yes ☐ No
Additional comments:____

Were you told you could keep your baby in the delivery room with you for an extended amount of time?
☐ Yes → Did you keep your baby with you for as long as you wanted? ☐ Yes ☐ No
Would you have preferred not to be offered this choice? ☐ Yes ☐ No
☐ No → Would this information have been helpful to you? ☐ Yes ☐ No
Additional comments:____

Were you told you could have photographs or video of your baby taken at birth?
☐ Yes → Did you get photographs or video of your baby? ☐ Yes ☐ No
Would you have preferred not to be offered this choice? ☐ Yes ☐ No
☐ No → Would this information have been helpful to you? ☐ Yes ☐ No
Additional comments:____

Were you told about the "Now I lay me down to sleep" complimentary photography program?
☐ Yes → Did you use "Now I lay me down to sleep"? ☐ Yes ☐ No
Would you have preferred not to be offered this choice? ☐ Yes ☐ No
☐ No → Would this information have been helpful to you? ☐ Yes ☐ No
Additional comments:____

Were you offered keepsakes of your baby such as handprints, footprints, or locks of hair?
☐ Yes → Did you ask for keepsakes such as handprints, footprints or locks of hair? ☐ Yes ☐ No
Would you have preferred not to be offered this choice? ☐ Yes ☐ No
☐ No → Would this have been helpful to you? ☐ Yes ☐ No
Additional comments:____

Were you offered the choice of who should cut the umbilical cord?
☐ Yes → Did you choose who should cut the cord? ☐ Yes ☐ No
Would you have preferred not to be offered this choice? ☐ Yes ☐ No
☐ No → Would this have been helpful to you? ☐ Yes ☐ No
Additional comments:____

Were you told you could bring in special clothing or a blanket for your baby?
☐ Yes → Did you bring in special clothing or a blanket? ☐ Yes ☐ No
Would you have preferred not to be offered this choice? ☐ Yes ☐ No
☐ No → Would this have been helpful to you? ☐ Yes ☐ No
Additional comments:____

Were you told you could bathe your baby?
☐ Yes → Did you choose to bathe your baby? ☐ Yes ☐ No
Would you have preferred not to be offered this choice? ☐ Yes ☐ No
☐ No → Would this have been helpful to you? ☐ Yes ☐ No
Additional comments:____

Were you asked about your special wishes for lighting in the room?
☐ Yes → Did you have special wishes for lighting in the room? ☐ Yes ☐ No
Would you have preferred not to be offered this choice? ☐ Yes ☐ No
☐ No → Would this have been helpful to you? ☐ Yes ☐ No
Additional comments:____

Were you asked about your special wishes for music in the room?
☐ Yes → Did you have special wishes for music in the room? ☐ Yes ☐ No
Would you have preferred not to be offered this choice? ☐ Yes ☐ No
☐ No → Would this have been helpful to you? ☐ Yes ☐ No
Additional comments:____

Were there any other services or options you were offered?
☐ Yes → Were these helpful for you? ☐ Yes ☐ No
☐ No

Are there any services or options that in retrospect you wish you had been offered? ____________________________
____

Were the number of options you were given for how you could spend time with your baby at birth
☐ Too many/too overwhelming
☐ Just right
☐ Not enough
Additional comments: ____

Did you know, before the baby was born, that there was a chance that he/she would not survive?
☐ Yes → Were private childbirth classes or caesarean section classes offered to you?
☐ Yes → Did you participate in such a class? ☐ Yes ☐ No
Would you have preferred not to be offered this choice? ☐ Yes ☐ No
☐ No → Would you have liked to have these classes offered to you? ☐ Yes ☐ No
☐ No
Additional comments: ____

Who is completing this survey?
☐ Mom
☐ Dad
☐ Other (please specify) ___________

Please feel free to use this space and, if necessary, additional paper for your comments:

NICU Survey

The following questions apply to the final days or hours of your baby's life:

In the final days or hours of your baby's life, from whom did you receive counseling regarding options for how you would like to spend the time you have with your baby? (Please check all that apply)

☐ Doctor/Nurse Practitioner in the NICU
☐ Nurse in the NICU
☐ Social Worker
☐ Hospital chaplain
☐ Other ____
☐ I did not receive counseling regarding options for how I would like to spend the time I have with my baby

Was it discussed with you that you could choose to have family members or other sources of support in your baby's room with you?

☐ Yes → Did you have a support person? ☐ Yes ☐ No
Would you have preferred not to be offered this choice? ☐ Yes ☐ No
☐ No → Would this information have been helpful? ☐ Yes ☐ No
Additional comments:____

Did your baby have older siblings?

☐ Yes → Was sibling visitation discussed with you? ☐ Yes → Did the sibling(s) visit? ☐ Yes ☐ No
Would you have preferred not to be offered this choice?
☐ Yes ☐ No
☐ No → Would this information have been helpful to you?
☐ Yes ☐ No
☐ No
Additional comments:____

Were you offered spiritual care? (Either someone from your own faith or a hospital-based chaplain?)

☐ Yes → Was a spiritual care giver present at any point during your baby's life? ☐ Yes ☐ No
Would you have preferred not to be offered this choice? ☐ Yes ☐ No
☐ No → Would this information have been helpful to you? ☐ Yes ☐ No
Additional comments:____

Do you identify yourself as religious?

☐ Yes
☐ No

Do you identify yourself as spiritual?

☐ Yes
☐ No

Were you told you could hold your baby?

☐ Yes → Did you hold your baby? ☐ Yes ☐ No
Would you have preferred not to be offered this choice? ☐ Yes ☐ No
☐ No → Would this information have been helpful to you? ☐ Yes ☐ No
Additional comments: ____

Were you told you could hold your undressed baby on your chest (skin-to-skin care) as a treatment option?
☐ Yes → Did you do skin-to-skin care with your baby? ☐ Yes ☐ No
Would you have preferred not to be offered this choice? ☐ Yes ☐ No
☐ No → Would this information have been helpful to you? ☐ Yes ☐ No
Additional comments:____

Were you told you could stay with your baby in his/her room for an extended amount of time after your baby passed away?
☐ Yes → Did you keep your baby with you for as long as you wanted? ☐ Yes ☐ No
Would you have preferred not to be offered this choice? ☐ Yes ☐ No
☐ No → Would this information have been helpful to you? ☐ Yes ☐ No
Additional comments:____

Were you told you could have photographs or video of your baby?
☐ Yes → Did you get photographs or video of your baby? ☐ Yes ☐ No
Would you have preferred not to be offered this choice? ☐ Yes ☐ No
☐ No → Would this information have been helpful to you? ☐ Yes ☐ No
Additional comments:____

Were you told about the "Now I lay me down to sleep" complimentary photography program?
☐ Yes → Did you use "Now I lay me down to sleep"? ☐ Yes ☐ No
Would you have preferred not to be offered this choice? ☐ Yes ☐ No
☐ No → Would this information have been helpful to you? ☐ Yes ☐ No
Additional comments:____

Were you offered keepsakes of your baby such as handprints, footprints, or locks of hair?
☐ Yes → Did you ask for keepsakes such as handprints, footprints or locks of hair? ☐ Yes ☐ No
Would you have preferred not to be offered this choice? ☐ Yes ☐ No
☐ No → Would this have been helpful to you? ☐ Yes ☐ No
Additional comments:____

Were you told you could bring in special clothing or a blanket for your baby?
☐ Yes → Did you bring in special clothing or a blanket? ☐ Yes ☐ No
Would you have preferred not to be offered this choice? ☐ Yes ☐ No
☐ No → Would this have been helpful to you? ☐ Yes ☐ No
Additional comments:____

Were you told you could bathe your baby?
☐ Yes → Did you choose to bathe your baby? ☐ Yes ☐ No
Would you have preferred not to be offered this choice? ☐ Yes ☐ No
☐ No → Would this have been helpful to you? ☐ Yes ☐ No
Additional comments:____

Were you asked about your special wishes for lighting in the room?
☐ Yes → Did you have special wishes for lighting in the room? ☐ Yes ☐ No
Would you have preferred not to be offered this choice? ☐ Yes ☐ No
☐ No → Would this have been helpful to you? ☐ Yes ☐ No
Additional comments:____

Were you asked about your special wishes for music in the room?
☐ Yes → Did you have special wishes for music in the room? ☐ Yes ☐ No
Would you have preferred not to be offered this choice? ☐ Yes ☐ No
☐ No → Would this have been helpful to you? ☐ Yes ☐ No
Additional comments:____

Were there any other services or options you were offered?
☐ Yes → Were these helpful for you? ☐ Yes ☐ No
☐ No

Are there any services or options that in retrospect you wish you had been offered? ____________________________
____

Were the number of options you were given for how you could spend time with your baby in his/her final days or hours
☐ Too many/too overwhelming
☐ Just right
☐ Not enough
Additional comments: ____

Did you know, before the baby was born, that there was a chance that he/she would not survive?
☐ Yes → Were private childbirth classes or caesarean section classes offered to you?
☐ Yes → Did you participate in such a class? ☐ Yes ☐ No
Would you have preferred not to be offered this choice? ☐ Yes ☐ No
☐ No → Would you have liked to have these classes offered to you? ☐ Yes ☐ No
☐ No
Additional comments: ____

Who is completing this survey?
☐ Mom
☐ Dad
☐ Other (please specify) __________

Please feel free to use this space and, if necessary, additional paper for your comments:
